# Health behaviour and COVID-19: Initial findings on the pandemic

**DOI:** 10.25646/7055

**Published:** 2020-11-18

**Authors:** Susanne Jordan, Anne Starker, Susanne Krug, Kristin Manz, Ramona Moosburger, Anja Schienkiewitz, Gianni Varnaccia, Johannes Zeiher, Benjamin Wachtler, Julika Loss

**Affiliations:** Robert Koch Institute, Berlin Department of Epidemiology and Health Monitoring

**Keywords:** TOBACCO, ALCOHOL, PHYSICAL ACTIVITY, NUTRITION, OBESITY, COVID-19, PANDEMIC, SARS-COV-2

## Abstract

The COVID-19 pandemic poses new challenges to both individuals and societies that impact health behaviour in many ways. This narrative review brings together initial findings for smoking, alcohol use, nutrition, physical activity and obesity. Smoking and obesity are potential direct risk factors for a severe course of COVID-19, and alcohol abuse, physical inactivity and an unbalanced diet can be indirect risk factors. The constraints of public life to contain the COVID-19 pandemic reduced the opportunities for physical activity and sports, although the initial results on physical activity during this period for Germany do not reflect this assumption. While a part of the population reports making healthier diet choices than before the pandemic, others do not. For smoking and risky alcohol use, data at an aggregate level for the general population do not indicate any behaviour changes. However, different trends appear to be emerging for different population groups pointing to the fact that social inequalities in pandemic-related changes to health behaviour must be assumed. Should further studies confirm these results, this would indicate a need for pandemic-specific prevention measures. Furthermore, specifically during the pandemic, prevention and health promotion measures directed at changes to health behaviour should continue to be implemented and adapted to the restrictions due to the pandemic. Equity in health should be promoted in particular.

## 1. Introduction

The COVID-19 pandemic has affected our behaviour in different ways, for example our interaction with other people or our online and purchasing behaviours. In particular, the measures to contain the COVID-19 pandemic were sometimes accompanied by major changes to our lifestyle and everyday behaviour. As there is no vaccination against a SARS-CoV-2 (Severe Acute Respiratory Syndrome Coronavirus 2) infection yet, protective measures mainly consist of non-pharmaceutical measures in form of behavioural recommendations and rules. Accordingly, to protect health, the population was asked to adopt behaviours such as reducing social contact, respecting physical distancing, wearing protective and everyday masks and to comply to hand hygiene rules [1, 2]. However, behaviours that are generally summarised under the term ‘health behaviour’ like healthy eating, exercise or avoiding the use of addictive substances, are also being discussed as influencing factors in the COVID-19 pandemic. For example, public life regulations to restrict people’s movement and contact with others (e.g. so-called lockdown) posed special challenges, which can also impact on the amount of exercise and nutritional behaviour and thus have a considerable influence on health and well-being. The regulations also closed access to sports facilities, restaurants and supermarkets or imposed barriers to such access.

People face different risks of contracting SARS-CoV-2 or developing a severe course of COVID-19. This fact has also drawn attention to health behaviour and its importance as a risk factor for contracting COVID-19 or developing a severe course of the disease. The factors considered specifically include smoking and obesity although alcohol use, lack of exercise and dietary habits are considered to be indirect factors.

**Figure 1:**
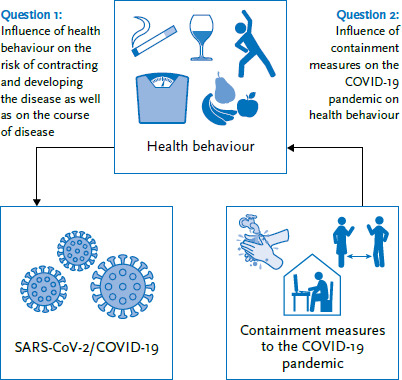
Questions considered by the paper Source: Own figure

This paper examines the role of health behaviour in COVID-19 infection and the potential changes to health behaviour caused by the measures taken to contain the COVID-19 pandemic (Figure 1). Smoking, alcohol use, nutrition, exercise and obesity were therefore the focus of the considerations in this paper.

## 2. Methodology

We conducted a narrative review to provide a broad overview of health behaviour in context of COVID-19 with the aim of providing a synopsis of the current state of research. The publications included in the review are based on research conducted between the end of February and mid-June 2020. Articles published after that date were not included. The literature search for health behaviour-related factors was conducted on the following databases and search engines: PubMed, Embase, Scopus, Cochrane Library, Google, Google Scholar, medRxiv (Preprint Server for Health Sciences), Novel Coronavirus Information Center (Elsevier’s free health and medical research on the novel coronavirus (SARS-CoV-2) and COVID-19), Qeios and ResearchGate COVID-19 Section. Titles and abstracts were searched for keywords like alcohol, smoking, physical activity, exercise, movement behaviour, restrictions, immune system, respiratory tract infections, diet, nutrition, obesity, covid, coronavirus and some German keywords such as körperliche Aktivität, Sport, Veränderung, Bewegungsverhalten, Einschränkungen, Atemwegsinfektionen. Inclusion criteria were German or English language and the availability of a full text. However, the selection of articles and sources was subjective and unsystematic and therefore does not claim to be complete and reproducible.

## 3. Results

### 3.1. Health behaviour and probability of contracting COVID-19 and severity of disease course

Regular smokers face higher risks for many infectious diseases and often also have a greater risk of suffering a more severe course of disease [3, 4]. In addition, with regard to COVID-19, smoking was discussed early on as a risk factor for a severe course of disease. Tobacco use also increases the risk of developing many noncommunicable diseases, such as chronic obstructive pulmonary disease (COPD) or diabetes mellitus, which are considered risk factors for a severe course of COVID-19 [5]. Numerous studies and several reviews have been published on the link between COVID-19 and tobacco use [6–13]. However, as the methodological quality of these studies was mostly poor, the significance of results is rather limited. There is currently no reliable evidence that SARS-CoV-2 infection rates among smokers are higher [12]. However, a correlation between tobacco use and the severity of COVID-19 is found: a regularly revised systematic literature review with meta-analysis concluded that current smoking is associated with a higher risk of severe COVID-19 disease progression than never smoking [12]. There is no statistically significant correlation between this risk and former smokers compared to people who never smoked. Statistically there is also no significant evidence of a higher mortality for smokers who develop a COVID-19 disease [12]. Preliminary initial evaluations of more than 5,000 deaths in England show that COVID-19 mortality was higher among former smokers (age-adjusted) [14].

Alcohol abuse (defined by monthly drinking frequency or amount of alcohol consumed per day) weakens the immune system [15] and can therefore be a relevant factor regarding COVID-19. So far no studies are known that investigate SARS-CoV-2 infections or (severe courses of) COVID-19 connected to levels of alcohol use. However, as a systematic review of 17 observational studies conducted between 1985 and 2015 shows [16], chronic alcohol abuse is a risk factor in acute respiratory distress syndrome (ARDS). ARDS is also one of the most serious COVID-19 complications [17, 18]. So far, however, no direct link between alcohol abuse or dependence and ARDS in COVID-19 has been described. On the contrary, for example on social media and other communication channels, the claim has been made that alcohol kills viruses and can thus prevent COVID-19. This is, however, not true. In a specific fact sheet on alcohol use and COVID-19, the World Health Organization (WHO) corrects this misinformation [19].

Physical inactivity is a risk factor for certain diseases that are being discussed in connection with a more severe course of COVID-19 [20]. These include obesity, type 2 diabetes and coronary heart disease. Thus, physical inactivity could indirectly – and over time – promote a more severe course of COVID-19. Only one study has investigated the potential correlation between physical inactivity and COVID-19 disease courses so far. It showed that a lack of physical activity increases the hospitalisation risk of COVID-19 patients [21]. Physical activity, on the other hand, can serve as a protective factor for various noncommunicable and chronic diseases [22]. Physical activity and doing sports also strengthen the immune system [23]. Regular light to moderate physical activity level can boost immune function and generally reduce the risk of contracting viral infections, as well as the duration or severity of the infection [24]. Yet, extremely strenuous physical activity during the early stages of an infection can increase the speed and make it easier for viruses to cross the upper airway immune barrier and so increase the risk of developing the disease [24].

At the time of the review, no scientific studies had investigated the effects of a healthy diet or the consumption of specific foods on a person’s risk of developing or suffering from a severe course of COVID-19. Ample evidence however indicates that a healthy and varied diet helps strengthen the immune system, where the interaction of many nutrients appears to play an important role, including vitamins A, C, D, and E and the trace elements zinc, selenium, iron and copper [25–29]. Studies on COVID-19 and nutrition have so far focused mainly on vitamin D. A protective effect of vitamin D supplementation in respiratory tract infections is also being discussed for COVID-19, especially regarding risk groups such as older people or people with stronger skin pigmentation [30–32]. Initial trials reported a lower vitamin D status (25(OH)D concentration in the blood) of COVID-19 patients and these patients were more likely to present a vitamin D deficiency than control groups [33, 34]. Some European and US-American observational studies have also described a tendency for COVID-19 mortality to be higher in countries where vitamin D deficiency is more common, yet where unable to provide evidence for a causal relationship [35, 36].

A balanced diet and sufficient physical activity make an important contribution to a proper energy balance, which can prevent a person from developing overweight and obesity. At the time of the literature review, no studies were found which indicated that obesity could increase the risk of a SARS-CoV-2 infection. However, preliminary analyses of patient data and data from cohort studies suggest that obesity can increase the risk of developing a COVID-19 disease [21, 37–39]. In addition, several clinical studies and first systematic reviews indicated that obesity can be a risk factor for a severe course of COVID-19 [21, 38–43]. This is mainly attributed to the fact that obesity is often associated with other diseases (e.g. diabetes mellitus, hypertension and lipometabolic disorders) [44, 45]. Furthermore, it is also assumed that, regardless of other risk factors, obesity itself may have an adverse effect on the course of COVID-19 [44, 45]. Possible mechanisms being discussed include metabolic changes caused by metabolic products of fat cells, which could have an impact on the functioning of the immune system, and therefore increasing the risk of a severe infection and reducing the effectiveness of antiviral drugs [44, 45]. Moreover, an increased fat mass – especially in the abdominal and thoracic part of the body – can impair normal respiratory function and thus worsen the course of a COVID-19 disease [44, 45]. Whether obesity among COVID-19 patients is associated with increased mortality is not yet clear [44]. However, there are indications that obesity could be associated with increased mortality due to COVID-19 [14, 46, 47].

In summary, smoking and obesity can be direct and chronic alcohol abuse, physical inactivity and an unbalanced diet can be indirect risk factors for a severe course of COVID-19. Health behaviours such as non-smoking, avoiding risky alcohol use, eating a balanced diet and sufficient physical activity help strengthen the immune system, which potentially positively affects the course of COVID-19 disease.

### 3.2 Health behaviour and measures to control COVID-19

Individual motivation and capability as well as the available options for action influence health behaviour and potential changes to health behaviour [48]. The restrictions imposed on public life to contain the COVID-19 pandemic have strongly influenced these options.

Public sphere restrictions have changed people’s habits regarding physical activity and sports. For example, sports courses did not take place and sports facilities were closed. Active commuting has been reduced by, among other things, working from home arrangements, closures of child care facilities and schools, and limited access to shopping and leisure facilities. The results of the COVID-19 Snapshot Monitoring (COSMO) [49], an online survey in Germany, which was conducted on 14 and 15 April 2020, did not show changes to leisure-related physical activity and muscle build-up exercise levels during the restrictive containment measures [49]. For the analysis, COSMO results were compared with those of the population-wide German Health Update (GEDA 2014/2015-EHIS) [50]. The results of the COSMO survey do not reveal the extent to which the overall level of physical activity including distances actively travelled has changed. A study from England confirms that adult population physical activity levels have remained stable [51]. However, an online cross-sectional survey on the training behaviour of the adult population in Belgium [52] revealed differences in physical activity behaviour: people in the age group 55 and older, those with low education and those who were used to exercising with friends or in a sports club stated that they had not exercised as much during lockdown. Overall, this study shows a general increase in training frequency as well as in sedentary behaviour in March 2020 compared to before. An analysis by the fitness wristband manufacturer Fitbit shows that people in Germany walked 11% fewer steps per day in March 2020 than in March 2019 [53]. At present, there is a lack of representative data to show physical activity levels for the different areas of life (work, leisure and transport) that would enable a differentiated evaluation (by age group, socioeconomic status and psychosocial issues). This data would be required to make detailed statements regarding the restrictive containment measures-related changes to activity levels across population groups.

The changes in everyday life resulting from the containment measures potentially also impacted on the dietary habits of the population. Initial findings have been published in various countries. However, the methodological quality of these studies is highly heterogeneous and usually accompanied by strong limitations such as a lacking claim of representativeness. Several cross-sectional studies from Europe and Australia indicate that COVID-19 related measures may have had a negative impact on the population’s dietary habits [54–59], for example a more frequent consumption of sweets and snacks [55–59]. In addition, many participants stated they had consumed less fresh fruit, vegetables and fish [57, 59]. A general increase in daily food consumption or an overall greater energy intake is also addressed by several studies [55, 56, 58, 59]. On the other hand, some cross-sectional studies do also report that the dietary habits of participants had become healthier during restrictive containment measures [57, 60]. For example, consumption of fruit and vegetables (fresh and frozen) and canned fish increased, while the consumption of soft drinks was lower than before [56, 57, 59]. The findings consistently point to increased cooking at home [57–59, 61]. Cohort data from the French NutriNet-Santé study also showed that some people’s eating habits had improved, while those of others had deteriorated or not changed at all [59]. The unfavourable changes to dietary behaviour included: more frequent consumption of snacks, less consumption of fresh foods (especially fish and fruit) and increased consumption of sweets, biscuits and cakes. Reported consumption changes of these foods in the opposite direction were considered to be favourable changes. The changes in dietary behaviour were associated with socioeconomic factors, and a person’s occupational and family situation. In the case of Germany, this year’s nutrition report shows initial data on nutritional behaviour during the COVID-19 pandemic. Around 30% of participants aged 14 and older stated that they had cooked more often themselves during the restrictive containment measures than before. In addition, people more often ate meals with their families than before the containment measures [61]. Overall, there are indications that different population groups have dealt differently with the restrictions imposed on daily routine by the containment measures, both in terms of dietary behaviour and physical activity.

Scant information is currently available on how measures to control the corona pandemic have affected the German population’s use of tobacco. Since smoking has been increasingly reported as a possible risk factor for a severe COVID-19 disease course, an increase in attempts to stop smoking seems plausible. At the same time, it is conceivable that social isolation, existential fears and health concerns could lead the use of tobacco products to rise, as many smokers experience consuming tobacco as a stress-reducing factor [62]. In addition, restriction of social contacts makes it more difficult to seek help to quit smoking [63]. An online survey on the use of alcohol and nicotine products during the restrictive containment measures revealed that 11% of smokers in Germany had stopped smoking [64]. On the other hand, 43% of the smokers interviewed answered that they had smoked more during the restrictive containment measures, while 9% reported they had smoked less. According to the study, specifically people with low education and those who experienced changes to employment conditions such as leave of absence or working from home arrangements increased their consumption. Results from England showed that a greater number of people had quit smoking or attempted to quit following restrictive containment measures [63]. In Germany, too, prevention measures included the suggestion to use the COVID-19 pandemic as an opportunity to quit smoking [65]. Here, further research needs to reveal how current prevention measures can be improved in the event of an epidemic or pandemic.

The COSMO survey provides data relating to alcohol use in Germany during the restrictive containment measures. However, this data does not show a trend towards more frequent alcohol use [49]. The Global Drug Survey (GDS), a worldwide online survey on drugs consumption, also provides results on changes in alcohol use during the COVID-19 pandemic. In a first interim evaluation, data from more than 40,000 people was analysed [66]. 44.0% of participants reported that they had drunk more alcohol during the pandemic, with 25.5% reporting that they had drunk less [66]. The main reasons are given for increased drinking are that people had more time to drink and often felt bored. About a third of respondents said that they had begun drinking earlier in the day than usual. The main reason for the decrease in alcohol use is said to be less social contact with people and settings where alcohol is commonly consumed. More than a third of those who drank less, reported improvements in physical health. Country-specific evaluations of the GDS are expected after the study is completed. The aforementioned online survey on consumption patterns for alcohol and nicotine products [64] showed that the majority of respondents (41.0%) had not reported changes to their alcohol use since the start of the restrictive containment measures. 21.2% reported a decrease and 37.2% an increase in consumption, while 0.4% said they had only taken up drinking alcohol during the restrictive containment measures. Results from England confirm an increase in risky alcohol use during restrictive containment measures, but also increased attempts to reduce alcohol use [63]. It also became clear that there were fewer services available to people during the COVID-19 pandemic.

Overall, not enough data is available on the effects of containment measures on health behaviour such as smoking, alcohol use, diet and physical activity. However, the evidence so far suggests that the impact of COVID-19 pandemic containment measures on different population groups varies. It is therefore necessary to carry out analyses of the potentially uneven impact of the measures taken. This makes it important and urgent to collect data for indicators of socioeconomic status and other social and lifestyle determinants of health behaviour and include them in the analyses.

## 4. Conclusion and outlook

The COVID-19 pandemic poses new challenges to people as individuals and as a society across the board, which affect health behaviour in many ways. Protection measures to contain the pandemic have at times demanded major lifestyle changes and are likely to affect our everyday health behaviour for some time. Restrictions or bans on social contact, temporary quarantine or physical distancing measures affect the opportunities people have to engage in physical activity, their shopping behaviour and working conditions, but also impact psychosocial health [67, 68]. From summer 2020, the extent to which future measures to contain the COVID-19 pandemic will restrict everyday life cannot yet be estimated. It is therefore crucial to understand the relationship between the COVID-19 pandemic and health behaviour in order to counteract with targeted prevention measures if necessary.

Initial data on physical activity behaviour for the German population indicate that people may not have been less physically active during restrictive containment measures than before, which is surprising given the initial restrictions and the closure of sports facilities. However, the only data available so far is from a non-representative online survey for adults. Moreover, sports clubs also began offering virtual exercise and other online courses [69]. Studies from other countries indicate that activity levels developed differently across population groups, for example, those used to exercise with friends or in a sports club now exercised less. Initial observations for dietary behaviour also indicate differentiated developments in the population. While some people reported taking healthier food choices, others seem to be eating less balanced as before the pandemic. For smoking and risky alcohol use, data at an aggregate level for the total population do not indicate any behaviour changes. However, inequalities between subgroups are emerging, as parts of the population increased their use of legal addictive substances during the restrictive containment measures, for example, more people with lower education smoked. Interestingly, for both alcohol and tobacco use, there is evidence that some people are consciously using the pandemic to drink less or quit smoking. It would seem important to provide targeted support to those wishing to reduce their consumption.

This narrative review has revealed the need to look more closely at health behaviour in the context of the COVID-19 pandemic as promoting healthier behaviour provides an opportunity to reduce the risk of a severe course of COVID-19 disease. To identify population groups whose health behaviour is particularly impacted by the effects of restrictive containment measures, for example people in difficult living conditions or with previous illnesses, will require population-wide studies. There is also a need for more precise knowledge about the extent of behavioural changes in different groups, categorised for example by gender, age and living conditions which will have to consider social determinants, sociodemographic factors and the setting (i.e. lived experience and social environment). Over and above indicators of socioeconomic status, this would have to include factors such as housing and working conditions or the settings that determine health behaviour (e.g. where and how physical activity was otherwise practised or where and how people ate). On the basis of such results, measures could be developed to support a healthy lifestyle under the special conditions of a pandemic, which could also be used during possible future epidemics. For example, (psychological) support measures could be developed, especially for the most vulnerable groups and for those who, as a result of the COVID-19 pandemic, developed an addiction or increase substance abuse (alcohol, tobacco) [70].

Should further studies corroborate these preliminary results, it would further underline the need for pandemic-specific accompanying prevention measures, particularly with regard to health equity. Setting-based prevention measures, which classically consist of a health-promoting redesign of living spaces such as communities, companies or schools, will, in part, have to be redesigned and/or adapted according to the measures to contain the COVID-19 pandemic. It is conceivable that health promotion could to a greater degree rely on increasing the use of public community spaces, if using inside spaces is associated to an increased risk of infection, or using digital technologies to involve the population in health promotion. Policies should also consider the pandemic’s economic impact, which as a social determinant, can influence health behaviour. The ‘Health in All Policies’ strategy can contribute to this. However, prevention aimed at changing individual health behaviour must also be adapted to the pandemic. An example of this is the special importance of risk communication in the context of a pandemic. It should motivate people to take informed decisions on protective health behaviour, e.g. through healthy eating or by observing physical distancing rules. In future, the development of strategies for the prevention of communicable and non-communicable diseases should go hand in hand.

The results presented here indicate that behaviours such as smoking, chronic alcohol abuse, physical inactivity, an unbalanced diet and obesity can be indirect or direct risk factors for a severe course of COVID-19. Further strengthening the evidence base regarding these correlations is important also against the backdrop of the increasing spread of myths and ‘fake news’ in the context of COVID-19 and health behaviour. Based on current knowledge, there is no food or dietary supplement that can protect anyone against a COVID-19 infection or help treat the disease. This also applies to nicotine and alcohol. In turn, a balanced diet, sufficient physical activity, refraining from smoking and avoiding risky alcohol use should be promoted as measures that strengthen the immune system. All in all, it is hoped that a better understanding of the correlations between lifestyle risk factors and COVID-19 will help both to prevent severe disease courses and to identify those most at risk.

Health behaviour in the context of the COVID-19 pandemic can be studied and assessed based on a wide array of research questions. The monitoring of different health-related behaviours in the general population under changing framework conditions during the pandemic – containment measures as well as social and economic consequences – is just as important as the identification of behaviour-related risk factors in COVID-19 patients. However, the pandemic has also drawn attention to the observance of rules in the sense of ‘new’ health behaviour such as physical distancing, paying attention to hygiene and wearing face masks, which play a decisive role in containing the pandemic. Knowledge gained from the promotion of healthy diets, physical activity or non-smoking (e.g. with regard to health communication, setting based prevention and structural public health measures) can be applied target group-specifically to promote COVID-19 protective behaviours.

## Key statements

- Smoking and obesity can be risk factors for a severe clinical course of COVID-19.
- A balanced diet and sufficient physical activity strengthen the immune system.
- Evidence on the effects of containment measures on health behaviour is scant and sometimes contradictory.
- The effects of containment measures differ in population groups.
- Research on COVID-19 and health behaviour should take social, sociodemographic and lifestyle-related factors into account.
